# *In vitro* and *in vivo* inhibitory effect of three Cox-2 inhibitors and epithelial-to-mesenchymal transition in human bladder cancer cell lines

**DOI:** 10.1038/bjc.2011.262

**Published:** 2011-07-12

**Authors:** Z Adhim, T Matsuoka, T Bito, K Shigemura, K-M Lee, M Kawabata, M Fujisawa, K Nibu, T Shirakawa

**Affiliations:** 1 Division of Infectious Disease Control, Center for Infectious Disease, Kobe University Graduate School of Medicine, 7-5-1, Kusunoki-cho, Chuo-ku, Kobe 650-0017, Japan; 2 Department of Otolaryngology Head and Neck Surgery, Kobe University, Graduate School of Medicine, Kobe, Japan; 3 Department of Clinical Molecular Medicine, Kobe University Graduate School of Medicine; 4 Division of Urology, Department of Organs Therapeutics, Kobe University Graduate School of Medicine, Kobe, Japan; 5 Department of Biochemistry, Korea University College of Medicine, Seoul, Republic of Korea

**Keywords:** Cox-2 inhibitor, E-cadherin, SNAIL, EMT, bladder cancer

## Abstract

**Background:**

Although the anti-tumour effect of cyclooxygenase-2 (Cox-2) inhibitors in invasive bladder cancer has been confirmed, its mechanisms of action are unclear. Recently, the concept of an epithelial-to-mesenchymal transition (EMT) promoting carcinoma progression has been suggested, and a key feature of the EMT is the downregulation of E-cadherin. In this study, we investigated the effect of Cox-2 inhibitors on reversal EMT and tumour growth inhibition in bladder cancer cells.

**Methods:**

We used three Cox-2 inhibitors, etodolac, celecoxib and NS-398 and three human bladder cancer cell lines, T24, 5637 and KK47, in this study. T24 xenograft tumour mouse model was used in the *in vivo* study.

**Results:**

Within the clinical drug concentrations, only etodolac showed the *in vitro* growth inhibition in T24 not in the other cell lines. Etodolac reduced *SNAIL* mRNA and vimentin cell surface expression, and induced *E-cadherin* mRNA and E-cadherin cell surface expression, in T24. Etodolac also most strongly inhibited the cell migration of T24 *in vitro* and showed the highest tumour growth inhibition in T24 tumour *in vivo*.

**Conclusion:**

Etodolac at clinical doses exhibited induced *in vitro* and *in vivo* anti-tumour effects and reversal effect of EMT in T24. These results suggest that etodolac is a good candidate for an anti-tumour or chemopreventive reagent for high-grade bladder cancer.

Bladder cancer, transitional cell carcinoma (TCC) of the urinary bladder, accounts for approximately 4.6% of all malignant tumour (Jemal *et al*, 2007). Although most cases of bladder cancer present as a superficial tumour, and are treated with transuretheral resection of the bladder tumour (TUR-BT), the recurrence rate after TUR-BT is high (30–85%) and approximately 10–30% of cases will progress to a muscle-invasive tumour that has a poorer prognosis (Pasin *et al*, 2008). Therefore, an effective strategy for preventing the progression of bladder cancer is clearly needed. Cyclooxygenase-2 (Cox-2) is overexpressed in high-grade invasive TCC, and the anti-tumour effect of Cox-2 inhibitors in invasive TCC of urinary bladder has been suggested in both pre-clinical and clinical studies (Okamoto *et al*, 2008; Parada *et al*, 2009; Qin *et al*, 2009; Dhawan *et al*, 2010). However, the mechanism of action for the anti-tumour effects of Cox-2 inhibitors is unclear.

Previously, we have reported that etodolac, a selective Cox-2 inhibitor, induced upregulation of E-cadherin and an *in vivo* growth inhibitory effect in high-grade human bladder cancer cell line T24 (Okamoto *et al*, 2008). E-cadherin, which is an epithelial cell adhesion molecule, is highly associated with tumour invasiveness, metastatic dissemination and poor prognosis (Guarino *et al*, 2007). There is a growing body of evidence suggesting that loss of E-cadherin expression or mutation in the *E-cadherin* gene may have a pivotal role in tumour progression as marked by increased mobility and invasiveness in various types of cancers, including bladder cancer (Birchmeier and Behrens, 1994; Imao *et al*, 1999).

Epithelial-to-mesenchymal transition (EMT) is the process by which epithelial cells dramatically alter their shape and motile behaviour as they differentiate into mesenchymal cells (Mendez *et al*, 2011). The most well-known familiar change that occurs during EMT is downregulation of surface E-cadherin expression, resulting in the loss of homotypic adhesion. During EMT, carcinoma cells become more motile, invasive and resistant to apoptosis by acquiring characteristics similar to embryonic mesenchymal cells, thereby allowing penetration of the stroma surrounding the initial neoplastic focus (Guarino *et al*, 2007). In contrast to E-cadherin, the epithelial marker of EMT, Zeb-1, Zeb-2 and vimentin are widely known as the mesenchymal markers of EMT (Sanchez-Tillo *et al*, 2010). McConkey *et al* (2009) measured these EMT markers in a panel of 20 human urothelial TCC cell lines and a set of 114 primary urothelial tumours, and observed a strong inverse correlation between the expression of E-cadherin and those of Zeb-1, Zeb-2 and vimentin. They found that the expression of the mesenchymal markers was confined to the muscle-invasive tumour. In addition, several previous studies have suggested that EMT was associated with bladder cancer progression and metastasis (Chaffer *et al*, 2006; McConkey *et al*, 2009; Kenney *et al*, 2011; Wiklund *et al*, 2011).

Both Cox-2 overexpression and the loss of E-cadherin expression are frequently detected in invasive bladder cancer (Shariat *et al*, 2001). Cox-2 and prostaglandin E2 expression have been associated with a significant reduction in E-cadherin and promotion of EMT via SNAIL, Zeb-1, Slug, Twist and other transcriptional factor-mediated mechanisms (Morgillo *et al*, 2007). In this study, we evaluated the *in vitro* and *in vivo* growth inhibitory effects of three different Cox-2 inhibitors, etodolac, celecoxib and NS-398 on three human bladder cancer cell lines, T24, 5637 and KK47, and we examined whether Cox-2 inhibitors could reverse the EMT in order to identify the mechanism of action for Cox-2 inhibitors as anti-tumour agents.

## Material and methods

### Cells and cell culture

The human urinary bladder cancer cell lines T24 (Bubenik *et al*, 1973) and 5637 (Fogh, 1978) were purchased from the American Type Culture Collection (Manassas, VA, USA); KK47 (Kotoh *et al*, 1997) was generously provided by Dr Naito (Kyushu University, Fukuoka, Japan). All cell lines were cultured in RPMI (Roswell Park Memorial Institute) 1640 medium (Gibco, Grand Island, NY, USA) supplemented with 10% fetal bovine serum, at 37°C with 5% carbon dioxide.

### *In vitro* cytotoxicity assay

The T24, 5637 and KK47 cells were seeded at a density of 500 per well in 96-well tissue culture plates. The etodolac was obtained from Nippon Shinyaku (Tokyo, Japan) and dissolved by dimethyl sulphoxide (DMSO; Sigma Chemical, St Louis, MO, USA). The celecoxib (4-(3-methyl-5-(4-methylphenyl)-1H-pyrazol-1-yl) benzenesulfonamide) was obtained from Key Organics (Camelford, UK) and NS-398 was obtained from Cayman Medical Company (Ann Arbor, MI, USA). All drugs were dissolved by DMSO. After 24 h of incubation, the cells were treated with etodolac, celecoxib and NS-398 at several concentration (at 10^−4^, 10^−5^, 10^−6^ or 10^−7^ M); the same volume of DMSO without a drug served as a control. The cell number was assessed at 2, 4 and 6 days after the initiation of drug treatment. The alamar blue assay was performed with a fluorimetric method according to the procedure described before (Ahmed *et al*, 1994). To determine the number of cells, the emitted fluorescence was compared with a standard curve from a known number of cells.

### Reverse transcriptase–PCR of mRNA expressions of Cox-2 and EMT-related markers

The cells were treated with 10^−5^ M concentrations of etodolac, celecoxib, NS-398 or DMSO (control) for 24 h, and then the cell pellets (5–10 × 10^−6^) were collected. Total RNA was extracted from each group using Trizol (Invitrogen, Carlsbad, CA, USA). The extracted mRNA was reverse transcribed using the Taq-Man Reverse Transcription Reagents kit (Applied Biosystems, Foster City, CA, USA). Real-time quantitative PCR using the SYBR Green I dye fluoresces system (Applied Biosystems) was performed for the relative quantification of the mRNA expression according to a previously described method (Guo *et al*, 2006). The sequences for the primers were as follows: *Cox-2*, forward: 5′-TGGACAGGGAGGATTTTGAG-3′, reverse: 5′-AGGCTGTGCCTTCCTACAGA-3′ *E-cadherin*, forward: 5′-ACGTCGTAATCACCACACTGA-3′, reverse: 5′-TTCGTCACTGCTACGTGTAGAA-3′ *Cytokeratin* 19, forward: 5′-GCGGGACAAGATTCTTGGTG-3′, reverse: 5′-CTTCAGGCCTTCGATCTGCAT-3′ *Vimentin*, forward: 5′-CTTCGCCAACTACATCGACA-3′, reverse: 5′-GCTTCAACGGCAAAGTTCTC-3′ *N-cadherin*, forward: 5′-ACAGTGGCCACCTACAAAGG-3′, reverse: 5′-CCGAGATGGGGTTGATAATG-3′ *SNAIL*, forward: 5-TCGTCCTTCTCCTCTACTTC-3′, reverse: 5-TTCCTTGTTGCAGTATTTGC-3′ *Slug*, forward: 5′-TCGGACCCACACATTACC-3′, reverse: 5′-CCGAGATGGGGTTGATAATG-3′ *Twist*, forward: 5′-AGCTGAGCAAGATTCAGACCCTC-3′, reverse: 5′-CCGTCTGGGAATCACTGT C-3′ *Zeb 1*, forward: 5′-CTGAAGAGGACCAGAGGCAG-3′, reverse: 5′-CCCAGAACTGCGTCACATGTC-3′ and *β-actin*, forward: 5′-GGACTTCGAGCAAGAGATGG-3′, reverse: 5-AGCACTGTGTTGGCGTACAG-3′. The PCR reactions were performed in the ABI7700 (Applied Biosystems) with PCR profiles as follows: 1 cycle for 5 min at 95°C, 45 cycles for 30 s at 94°C, 30 s at 56°C, 1 min at 72°C and with final cooling to 40°C. The values of *β-actin* mRNA were used as an endogenous control to normalise for differences in the amount of total RNA.

### Flow cytometric analysis of E-cadherin and vimentin expression

E-cadherin and vimentin expression in three different cell lines, treated with etodolac, celecoxib or NS-398 at 10^−5^ M or DMSO alone for 36 h. Cells were harvested by a short trypsinization of confluent monolayers. Cell suspensions were made in phosphate-buffered saline (PBS) at a concentration of 10^6^ cells ml^−1^. They were blocked by incubating the cell suspension with 1 *μ*g of Affinity Purified anti-mouse CD16/32 – blocks Fc binding (eBioscience Inc., San Diego, CA, USA) for 10 min, followed by normal mouse IgG_1_ (Santa Cruz Biotechnology Inc., Santa Cruz, CA, USA) as a negative control and 1 *μ*g of E-cadherin unconjugated primary antibodies (Santa Cruz Biotechnology Inc.) per 100 *μ*l of the prepared cell suspension (equivalent to one million cells) at 4°C for 30 min. To wash off excess antibody following staining, we added 2 ml of 1 × PBS to each tube, centrifuged them for 5 min at 2000 r.p.m., and aspirated the supernatant. The cell suspension was incubated in Alexa Fluor 647 goat anti-mouse IgG (Invitrogen) at 4°C for 30 min, washed with PBS and fixed in 2% paraformaldehyde. For the vimentin expression analysis, we used vimentin-conjugated FITC primary antibodies (Santa Cruz Biotechnology Inc.), and performed the cell permeabilisation before analysis. Data acquisition and analysis were performed on duplicate samples on a FACScan flow cytometer (FCM) using CELLQuest software (Becton Dickinson, San Jose, CA, USA).

### Immunocytofluorescence staining

T24 cell lines for immunocytofluorescence staining of E-cadherin and vimentin were plated on glass coverslips in four-well culture dishes and treated with 10^−5^ M concentrations of etodolac, celecoxib, NS-398 or DMSO. At 72 h after the initiation of drug treatment, were washed with PBS three times and fixed with cold methanol for 10 min on ice and permeabilised in 0.5% Triton X-100/PBS for 5 min at room temperature. After washing with PBS for 5 min (three times), cells were blocked with normal goat serum 10% for 30 min at room temperature, and then incubated with the specific primary antibodies. The following primary antibodies were used for immunostaining: E-cadherin unconjugated primary antibodies (Santa Cruz Biotechnology Inc.) at 1 : 200 dilution in PBS and vimentin conjugated FITC primary antibodies (Santa Cruz Biotechnology Inc.) at 1 : 100 dilution in PBS. Alexa fluor-647 (Invitrogen) conjugated secondary antibodies for E-cadherin were used. Cells were co-stained with 4′,6-diamidino-2-phenylindole (Molecular Probes, Eugene, OR, USA) at 1 : 500 dilution in PBS, to visualise the nuclei. Stained cells were mounted with fluorescent mounting medium (Dako, Carpinteria, CA, USA). The fluorescent images were obtained by confocal laser scanning microscope (LSM 700; Carl Zeiss Meditec, Göttingen, Germany).

### Wound healing assay

We assessed cell migration by determining the ability of the cells to move into an acellular space in a two-dimensional *in vitro* wound healing assay. Confluent cell monolayers were wounded by manually scraping the cells with a pipette tip. Debris was removed from the culture by washing twice with PBS. After that the cells were incubated with medium including FBS 10% and drug (etodolac, celecoxib, NS-398 at 10^−5^ M or DMSO as the control). Images were acquired immediately at 0 h (baseline control) and at 24 h using a BIOREVO BZ-9000 microscope (Keyence, Osaka, Japan). The wound area without cells was calculated using VH software Keyence (Keyence), and we then compared the total wound area without cells after 24 h of exposure of each drug.

### Animal experiments

In all, 24 athymic BALB/c (nu/nu) mice (5 weeks old) were purchased from Charles River Japan (Yokohama, Japan). T24 cells (5.0 × 10^6^) suspended in 250 *μ*l of RPMI 1640 mixed with 250 *μ*l BD Matrigel (Becton Dickinson, Franklin Lakes, NJ, USA) were transplanted to the backs of these mice. At 14 days after tumour inoculation, the T24 animals were randomly assigned to four experimental groups of six animal each as follows: group 1, PBS (+DMSO) treatment control group (six animals); group 2, etodolac-treatment group; group 3, celecoxib-treatment group; group 4, NS-398-treatment group. Beginning at 14 days after tumour inoculation, the tumour size was measured every other day and was calculated using the following formula: volume (a rotational ellipsoid)=M1 × M2^2^ × 0.5236, where M1=the long axis and M2=the short axis. These mice were intraperitoneally injected with etodolac (10 mg kg^−1^ body weight per day), celecoxib (10 mg kg^−1^ body weight per day), NS-398 (10 mg kg^−1^ body weight per day) or PBS+ DMSO using the same volume as with the drugs daily for 4 weeks, using a microliter syringe fitted with a 28-gauge needle. The tumours were extracted at 4 weeks after treatment initiation for apoptosis analysis. All procedures involving the mice were approved by the Institutional Animal Care and Use Committee (Permission No. P070802), and performed according to the Guidelines of Animal Experimentation of Kobe University.

### TUNEL assay

The TUNEL assay (Takara Bio Inc., Shiga, Japan) method uses terminal deoxynucleotidyl transferase to label the 3′-OH ends of DNA fragments that are generated during the process of apoptosis. The cells undergoing apoptosis are specifically labelled with fluorescein–dUTP with high sensitivity, allowing their immediate detection by viewing with a BIOREVO BZ-9000 fluorescence microscope (Keyence).

To quantitate apoptotic-positive cells per eight random slide was estimated by processing × 80 images using Image J 1.41 software (National Institutes of Health, Bethesda, MD, USA). Apoptotic index was standardised by that of control group being as 1 and expressed as the arbitrary unit.

### Statistical analysis

Determination of statistical significance was performed using a *t*-test for direct two group comparisons and analysis of variance for multiple group comparisons. All data are reported as s.d. is an index of the variability of the original data points and reported as ±s.e.m. if the study were repeated of three independent experiments. Statistical significance was set at *P*<0.05.

## Results

### Celecoxib and NS-398 inhibited the cell growth of all the three cell lines, but etodolac inhibited only growth of only T24 and 5637

**Figure 1:**
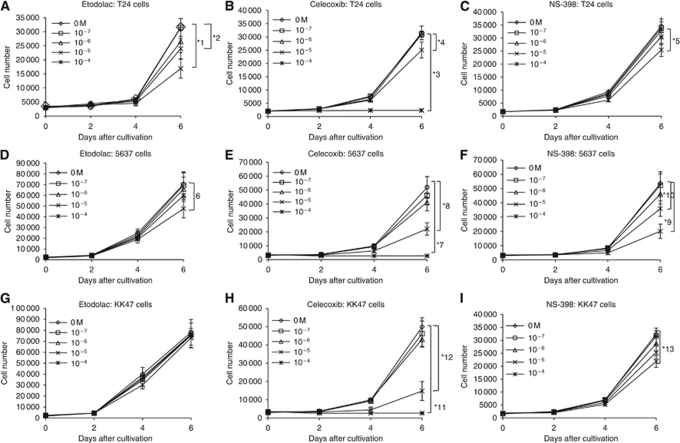
*In vitro* cytotoxicity in T24 (**A**–**C**), 5637 (**D**–**F**) and KK47 (**G**–**I**) cell lines treated with etodolac, celecoxib and NS-398. All significant differences at day 6 were marked with asterisks (^*^) 1–13. Each point represents triplicate averages, with ±s.e.m. bars. ^*^1, *P*=0.008; ^*^2, *P*<0.005; ^*^3, *P*<0.0001; ^*^4, *P*<0.025; ^*^5, *P*=0.004; ^*^6, *P*=0.0002; ^*^7, *P*=0.0001; ^*^8, *P*=0.001; ^*^9, *P*=0.019; ^*^10, *P*=0.013; ^*^11, *P*<0.001; ^*^12, *P*=0.001; ^*^13, *P*<0.0001.

The three human bladder cancer cell lines; T24, 5637 and KK47, originated from tumours of three different histological grades: III, II and I (Bubenik *et al*, 1973; Kotoh *et al*, 1997). The significant cell growth inhibitory effect compared with control (0 M) was observed in T24 cells with 10^−4^–10^−5^ M of etodolac and celecoxib, and 10^−4^ M of NS-398, in 5637 cells with 10^4^ M of etodolac, and 10^−4^–10^−5^ M of celecoxib and NS-398, and in KK47 cells with 10^−4^–10^−5^ M of celecoxib and 10^−5^ M of NS-398 (Figure 1G–I).

### T24 cells expressed the lowest level of E-cadherin and cytokeratin, and the highest level of Cox-2, vimentin and transcriptional factors (SNAIL, Slug, Twist and Zeb 1) among the three cell lines

**Figure 2:**
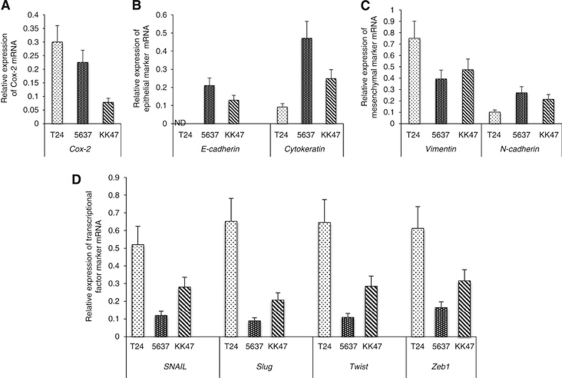
Relative mRNA levels of (**A**) *Cox-2*, (**B**) epithelial marker (*E-cadherin* and *Cytokeratin*) (**C**) mesenchymal marker (*Vimentin* and *N-cadherin*) and (**D**) transcriptional factor (*SNAIL*, *slug*, *twist* and *Zeb 1*) in the three bladder cancer cell lines. T24 cells expressed the highest *Cox-2*, lowest *E-cadherin and cytokeratin*, highest *Cox-2*, *vimentin* and transcriptional factor (*SNAIL*, *slug*, *twist* and *Zeb 1*) mRNA among three cell lines. Each point represent triplicate averages with ±s.e.m. bars. *β-Actin* was used as the endogenous RNA control to normalise for differences in the amount of total RNA.

To characterise the EMT features of the three cell lines, we compared the mRNA expressions of *Cox-2*, epithelial markers (*E-cadherin*, *Cytokeratin*), mesenchymal markers (*Vimentin*, *N-cadherin*) and transcriptional factors (*SNAIL*, *Slug*, *Twist* and *Zeb-1*), which are key signal factors of EMT in each cell line. Consistent with previous studies, we found that T24 cells originating from the highest grade III TCC expressed the lowest level of *E-Cadherin* and *Cytokeratin*, and the highest level of *Cox-2*, *Vimentin* and EMT-transcriptional factors (*SNAIL*, *Slug*, *Twist* and *Zeb 1*) mRNA among the three human bladder cancer cell lines (Figure 2). In addition, we found an inverse correlation between epithelial markers and mesenchymal markers and transcriptional factors mRNA expressions, that is, the lowest *E-cadherin* and the highest *Vimentin* and *SNAIL* were found in T24 cells and the highest *E-cadherin* and the lowest *Vimentin* and *SNAIL* were found in 5637 cells (Figure 2). These findings suggest the strongest EMT features in the T24 cell line.

### Cox-2 inhibitors enhanced mRNA expressions of epithelial markers and suppressed mRNA expressions of mesenchymal markers and EMT transcriptional factors in all the three cell lines

**Figure 3:**
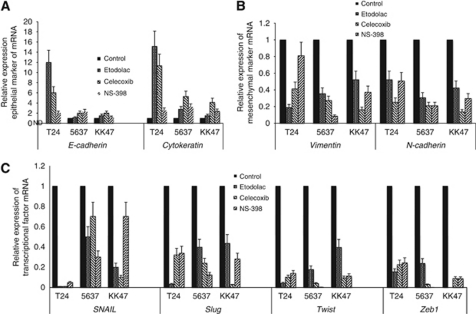
Relative mRNA levels of epithelial marker (*E-cadherin* and *cytokeratin*) (**A**), mesenchymal marker (*vimentin* and *N-cadherin*) (**B**), and transcriptional factor (*SNAIL*, *slug*, *twist* and *Zeb 1*) (**C**) in T24, 5637 and KK47 with or without Cox-2 inhibitors at 10^5^ M. Each point represent triplicate averages, with ±s.e.m. bars. *β*-Actin was used as the endogenous RNA control to normalise for differences in the amount of total RNA.

To investigate the effect of *Cox-2* inhibitors on EMT in three human bladder cancer cell lines, we examined the mRNA expressions of epithelial markers (*E-cadherin* and *Cytokeratin*), mesenchymal markers (*Vimentin* and *N-cadherin*) and EMT-transcriptional factors (*SNAIL*, *Slug*, *Twist* and *Zeb 1*) in the cell lines with or without Cox-2 inhibitors. Etodolac significantly enhanced *E-cadherin* mRNA in the T24 and KK47 cell lines, *Cytokeratin* in all three cell lines; celecoxib significantly enhanced *E-cadherin* and *Cytokeratin* mRNA in all three cell lines; and NS-398 enhanced *E-cadherin* mRNA in the T24 and 5637 cell line, *Cytokeratin* in all three cell lines. In the T24 cell line, etodolac induced the highest expression of *E-cadherin* mRNA compared with the other Cox-2 inhibitors (Figure 3A). All Cox-2 inhibitors significantly suppressed the mRNA expression of mesenchymal markers and EMT transcriptional factors.

### Etodolac most strongly induced E-cadherin expression and reduced vimentin expression on the T24 cell surface

**Figure 4:**
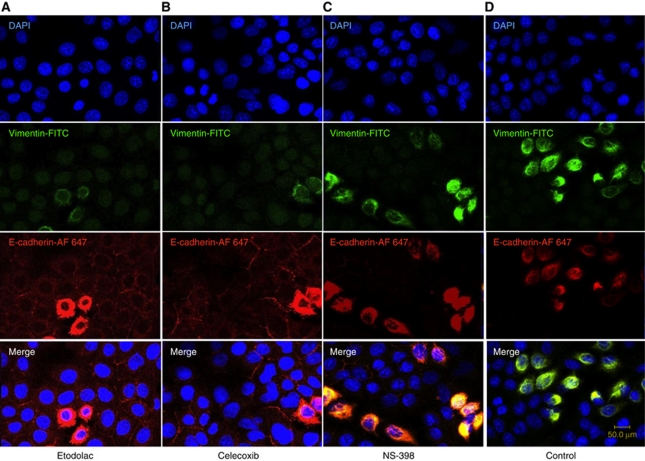
Etodolac and celecoxib but not NS-398 induced the mesenchymal-to-epithelial transition type changes in T24 cell lines. Immunofluorescence staining for DAPI stained nuclei blue, E-cadherin–Alexa Flour 647 (red) and vimentin-FITC (green) in T24 cell lines were evaluated by confocal laser microscopy. Vimentin staining was observed in cytoskeletal lesion of control and NS-398-treated cells (**C**, **D**), but not in that of etodolac- and celecoxib-treated cells (**A**, **B**). E-cadherin expressions were observed in cell surfaces of etodolac- and celecoxib-treated cells (**A**, **B**). Although control and NS-398-treated cells showed the mesenchymal features of cell appearance with losing cell–cell contact and an elongated phenotype (**C**, **D**), etodolac- and celecoxib-treated cells showed the characteristic ‘cobblestone’ appearance of epithelial cells (**A**, **D**). Scale bar, 50 *μ*m. The colour reproduction of this figure is available at the *British Journal of Cancer* online.

We also examined the cell surface markers of EMT: E-cadherin as an epithelial marker and vimentin as a mesenchymal marker. We calculated the changes in the ratio of E-cadherin or vimentin expressed cells: A–B/B: A=marker expressed cell number with Cox-2 inhibitors, B=marker expressed cell number without Cox-2 inhibitors. In the T24 cell line, etodolac most strongly increased E-cadherin cell surface expression (+21.62%) and decreased vimentin cell-surface expression (−20.19% Figure 4A and D). In the 5637 cell line, NS-398 most strongly increased E-cadherin cell surface expression (+17.45%) and decreased vimentin cell surface expression (−18.98% Figure 4B and E). In the KK47 cell line, celecoxib most strongly increased E-cadherin cell surface expression (+14.43%) and decreased vimentin cell surface expression (−14.73% Figure 4C and F). Interestingly, the Cox-2 inhibitor that most strongly decreased vimentin most strongly increased E-cadherin in all cell lines. In addition, the greatest increase in the ratio of *E-cadherin* expression and the greatest decrease in the ratio of vimentin expression were induced by etodolac in the T24 cell line.

### Etodolac and celecoxib but not NS-398 induced the mesenchymal-to-epithelial (MET) transition type changes in T24 cells

**Figure 5:**
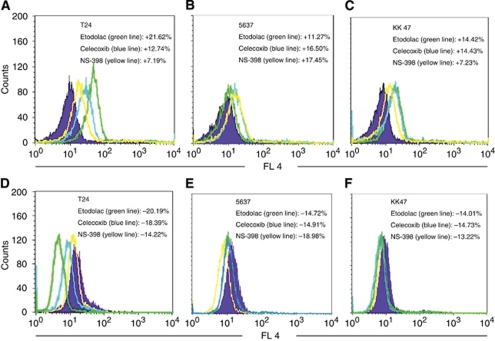
FCM analysis of E-cadherin (**A**–**C**) and vimentin (**D**–**F**) after treatment with DMSO as a control, etodolac, celecoxib and NS 398 at 10^5^ M on T24, KK47 and NS398 cell lines. Results were given as the percentage of up or downregulatian of E-cadherin and vimentin. We calculated the changes in the ratio of E-cadherin or vimentin expressed cells: A–B/B: A=marker expressed cell number with Cox-2 inhibitors, B=marker expressed cell number without Cox-2 inhibitors. In the histograms, the colour lines represent staining with E-cadherin or vimentin antibody after treatment with Cox-2 inhibitors, and the purple histogram represents the control without Cox-2 inhibitors. The colour reproduction of this figure is available at the *British Journal of Cancer* online.

An inverse correlation between E-cadherin and vimentin was observed across the treatment groups. Vimentin staining was observed in cytoskeletal lesion of control and NS-398-treated cells (Figure 5C and D), but not in that of etodolac- and celecoxib-treated cells (Figure 5A and B). Etodolac and celecoxib induced E-cadherin expressions in cell surfaces (intercellular membrane) of T24 cells (Figure 5A and B). In addition, although control and NS-398-treated cells showed the mesenchymal features of cell appearance with losing cell–cell contact and an elongated phenotype (Figure 5C and D), etodolac- and celecoxib-treated cells showed the characteristic ‘cobblestone’ appearance of epithelial cells (Figure 5A and B).

### Etodolac suppressed cell migration in T24 cell line

**Figure 6:**
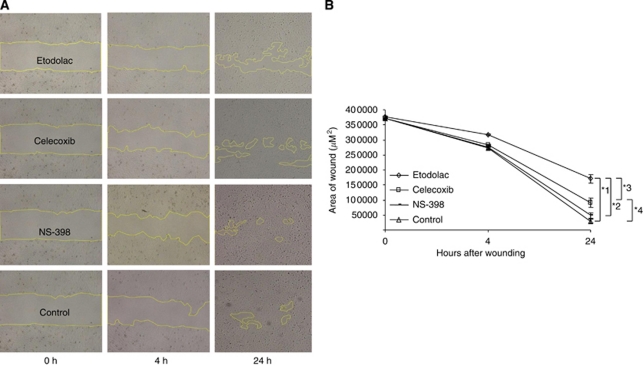
T24 cells were incubated with medium with Cox-2 inhibitors at 10^−5^ M and allowed to migrate into wound area for up to 24 h at 37°C. Images were acquired immediately, 0, 4 and 24 h. Wound area without cells was calculated and then we compared the total wound area without cell of each drug (**A**). At 24 h after creating scratch, T-24 cells treated with Cox-2 inhibitors migrated slower than the control cells. The highest inhibition of cell migration was observed in the cells treated with etodolac in 24 h (**B**). Each point represents the triplicate average of the area without cells of each treatment drug with ±s.e.m. bars. ^*^1, *P*<0.0001; ^*^2, *P*=0.0012; ^*^3, *P*=0.01; ^*^4, *P*=0.002.

The T24 cell line was isolated from the highest histological grade III tumour, and expressed the lowest level of *E-cadherin* among the three bladder cancer cell lines used in this study. Cells that undergo EMT shows enhanced cell migration as a mesenchymal feature. To investigate the effect of Cox-2 inhibitors on the cell migration of T24 cells, we performed an *in vitro* wound healing assay, which is commonly used for assessing the effect of pro- and anti-migratory agents on culturing cells (Raftopoulou *et al*, 2004). As shown in Figure 6A, 24 h after creating the wound, T-24 cells treated with Cox-2 inhibitors migrated slower than the control cells. The highest inhibition of cell migration was observed in the cells treated with etodolac after 24 h (Figure 6B).

### Etodolac significantly suppressed T24 tumour growth *in vivo*

**Figure 7:**
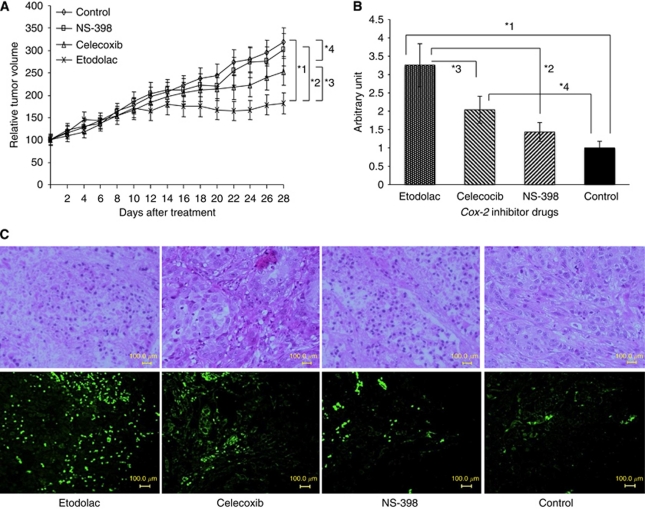
*In vivo* tumour growth curve (**A**) and TUNEL staining (**B**, **C**) of T24 tumour treated with Cox-2 inhibitors. (**A**) The daily administration of 10 mg kg^−1^ body weight per day of etodolac significantly suppressed the tumour growth at weeks 3 and 4 compared with that of the celecoxib, NS-398 and DMSO treatment group. The relative tumour volume was calculated assuming the rate of each tumour volume at day 0 to be 100. Each point represents the average of the tumours of each treatment group, with ±s.d. bars. ^*^1, *P*=0.031; ^*^2, *P*=0.05; ^*^3, *P*=0.04; ^*^4, *P*=0.002. (**B**) Apoptotic cells detected by TUNEL staining of tumour tissues. Apoptotic index of each group was expressed as its relative proportion to the untreated control. Each point represents the average of the tumours of each treatment group, with ±s.d. bars. Magnification × 80. Statistical significance was evaluated by Student's *t*-test. The difference in apoptotic index among some treatment group and control reached statistical significance (^*^1, *P*<0.0001; ^*^2, *P*=0.001; ^*^3, *P*=0.03; ^*^4, *P*=0.024, respectively). (**C**) The TUNEL-positive cells in the Cox-2 inhibitor treatment groups, etodolac, celecoxib and NS-398 but it was minimal in those in the control group.

We examined the *in vivo* growth inhibitory effect of Cox-2 inhibitors on subcutaneous tumours of the most invasive T24 cell line. Although the *in vitro* study showed that celecoxib had a higher inhibitory effect on T24 cell growth than did etodolac, the *in vivo* study showed that etodolac most highly inhibited the T24 tumour growth among the three Cox-2 inhibitors (Figure 7A).

### Cox-2 inhibitors induced cell apoptosis in T24 tumour *in vivo*

One of the mesenchymal features of the cells undergoing EMT is the acquisition of resistance to cell apoptosis. To investigate whether Cox-2 inhibitors could induce cell apoptosis, we perform a TUNEL staining assay for T24 tumour specimens isolated from the *in vivo* study. As shown in Figure 7B, in all group, significantly increased of apoptotic index was observed along etodolac group with celecoxib, NS-398 and control group (*P*=0.03, *P*=0.001, *P*<0.0001, respectively) and celecoxib group with control group (*P*=0.024). We observed the TUNEL-positive cells in the Cox-2 inhibitor treatment groups, but few were observed in the control group (Figure 7C), suggesting that Cox-2 inhibitors could induce cell apoptosis in T24 tumours *in vivo*.

## Discussion

In our *in vitro* study, both 10^−4^ and 10^−5^ M of celecoxib significantly inhibited the cell growth of all the three bladder cancer cell lines, but 10^−5^ M of etodolac or NS-398 significantly inhibited only T24 or 5637 cell line. These finding suggest that celecoxib had the strongest anti-tumour effect in bladder cancer cell lines. However, the interpretation of *in vitro* studies for an anti-tumour effect of Cox-2 inhibitors is complicated with regard to the drug concentration. Previous studies reported that much higher concentrations of celecoxib than those attainable in the serum concentration at the approved clinical dosage were required to inhibit cell growth of human bladder cancer cells (Ferrandina *et al*, 2003; Patel *et al*, 2005). Celecoxib and etodolac are commercially available in many countries, and both drugs are commonly administered with a dosage of 200 mg two or three times a day. However, the maximum plasma concentrations of celecoxib and etodolac after oral administration at this dosage in humans were 2.14 × 10^−6^ and 4.25 × 10^−5^ M (Kuriyama *et al*, 1987; Williams *et al*, 2000). With the concentrations in our *in vitro* study, only 10^−5^ M of etodolac achieved the *in vitro* growth inhibitory effect in T24 cells within the clinical drug concentration. However, Williams *et al* (2000) demonstrated that 2.3 × 10^−6^ M of celecoxib, which could not achieve the *in vitro* cell growth inhibitory effect, significantly reduced the *in vivo* tumour growth of HCA-7, a human colon cancer cell line, in nude mice xenografts. This discrepancy between *in vitro* and *in vivo* studies suggest the involvement of factors other than the anti-proliferative effect of Cox-2 inhibitors.

Recent studies have shown that EMT has crucial roles in not only embryonic development and tissue repair but also in the progression of carcinoma (Hugo *et al*, 2007; Wu and Zhou, 2008; Yang and Weinberg, 2008). EMT facilitates cell migration and invasion, induces stem cell properties, prevents apoptosis and senescence, and contributes to immunosuppression. Thus, EMT is involved in many critical events during tumour metastasis and progression. In this study, we examined whether Cox-2 inhibitors could induce MET, which is the reversal of EMT, in three human bladder cancer cell lines.

The loss of E-cadherin is a fundamental event in EMT and is induced by E-cadherin repressors such as SNAIL, Zeb, Slug, Twist and others (Onder *et al*, 2008). *SNAIL* binds to E-box consensus sequences in the *E-cadherin* promoter and tightly regulates the *E-cadherin* expression at the transcriptional level (Cano *et al*, 2000). Among the three human bladder cancer cell lines, T24, 5637 and KK47, originated from tumours of three different histological grades: III, II and I (Bubenik *et al*, 1973; Kotoh *et al*, 1997), T24 showed the strongest EMT feature, characterised by loss of mRNA expressions of *E-cadherin* and *cytokeratin*, and overexpressions of mRNA of *vimentin*, *SNAIL*, *Slug*, *Twist* and *Zeb1* (Figure 2). T24 cells also expressed the highest level of *Cox-2* mRNA and were originally isolated from the invasive grade III TCC of the urinary bladder. The strong inverse correlations between the expressions of *E-cadherin* and E-cadherin repressors, also called EMT transcriptional factors, *SNAIL*, *Slug*, *Twist* and *Zeb1*, were confirmed in the quantitative PCR study, which also showed that the highest *E-cadherin* expression and the lowest expressions of EMT transcriptional factors in 5637 cells (Figure 2). Interestingly, each of the three Cox-2 inhibitors at 10^−5^ M showed the highest induction of *E-cadherin* and suppression of the most of EMT transcriptional factors across all cell lines. Etodolac most strongly induced the expression of *E-cadherin* and suppressed the expression of *SNAIL*, *Slug*, *Twist* and *Zeb1*, in T24, NS-398 most strongly induced *E-cadherin* and suppressed the *SNAIL*, *Slug*, *Twist* and *Zeb1*, in 5637, and celecoxib most strongly induced the expression of *E-cadherin* and suppressed the expression of *SNAIL*, *Slug* and *Twist*, in KK47 (Figure 3A and C).

We also examined the cell surface expression of E-cadherin, as an epithelial marker, and vimentin, as a mesenchymal marker, in the three bladder cancer cell lines treated with the three Cox-2 inhibitors using the FCM. Consistent with the quantitative PCR study for EMT molecular signal markers, the *E-cadherin* and *SNAIL* genes, etodolac most strongly induced E-cadherin expression and reduced vimentin expression on the cell surface of T24, NS-398 most strongly induced E-cadherin and reduced *vimentin* on 5637, and celecoxib most strongly induced E-cadherin and reduced *vimentin* on KK47 (Figure 4). These drug affinities to each cell line should be further investigated to elucidate the mechanism of action for anti-tumour activity of Cox-2 inhibitors. Previously, Kuriyama *et al* (1987) measured the IC50 values for Cox-1 and Cox-2 of different Cox-2 inhibitors by using human peripheral monocytes. Kato *et al* (2001) reported that the respective mean IC50 values for Cox-1 and Cox-2 IC50 (10^−6^ M), and the Cox-1/Cox-2 ratio of each drug were as follows: celecoxib, 82, 6.8, 12; etodolac, >100, 53, >1.9; NS-398, 125, 5.6, 22. Indeed etodolac has the lowest Cox-2 selectivity and highest Cox-2 IC50 (5.3 × 10^−5^ M) among the three COX-2 inhibitors in their experiments. From these data, Cox-2 inhibition might not directly contribute to the *in vivo* tumour growth inhibitory effect and reversal EMT effect in human bladder cancer cells that we used here.

Recent investigations have suggested that EMT has essential roles in tumour invasion and metastasis, and which are among the most characteristic features of aggressive cancer cells (Sabbah *et al*, 2008; Thiery, 2009). The mesenchymal properties of EMT are reported to reduce adhesion and enhance migration of cancer cells, and to promote both the tumour invasion and metastasis (Thiery, 2002). Our immunofluorescence staining study (Figure 5) revealed that etodolac and celecoxib enhanced the expression of E-cadherin in intracellular membrane, suppressed the expression of vimentin in cytoskeketal lesion, and induced the characteristic ‘cobblestone’ appearance of epithelial cells (Figure 5A and B), but NS-398 did not induce these changes in T24 cells (Figure 5C). We also evaluated the effect of the three Cox-2 inhibitors on the cell migration of the most invasive T24 cell line, using the wound healing assay, and found that etodolac at 10^−5^ M most strongly inhibited the cell migration of T24 compared with the other Cox-2 inhibitors (Figure 6). In the invasive T24 cell line, etodolac markedly reduced *SNAIL* mRNA expression and the vimentin cell surface expression, whereas it induced *E-cadherin* mRNA expression and E-cadherin cell surface expression. It also strongly inhibited cell migration. Taken together, these data suggest that etodolac might have a reversal effect on EMT in the T24 cell line. In the present *in vivo* study, etodolac also showed the highest tumour growth inhibitory effect in T24 xenograft tumour model compared with the other Cox-2 inhibitors (Figure 7A). In addition, we observed the significant increase of the cell apoptosis in the tumour tissues treated with etodolac and celecoxib compared with control, and etodolac compared with the other groups (Figure 7B and D). Furthermore, the dose (10 mg kg^−1^ body weight per day) of the three Cox-2 inhibitors used in this *in vivo* experiment was close to that commonly used in clinical applications for analgesia 400–600 mg per day (Lin *et al*, 2006; Okamoto *et al*, 2008).

Although selective Cox-2 inhibitors have significantly fewer gastrointestinal side-effects compared with traditional non-steroidal anti-inflammatory drugs, many reports have been published on the potential cardiovascular and thromboembolic complications of higher dose of celecoxib (Solomon *et al*, 2005, 2006). Thus, it is critical to achieve an anti-tumour effect with an acceptable dose of Cox-2 inhibitors. In the present study, we demonstrated that the standard clinical drug concentrations of etodolac could induce both *in vitro* and *in vivo* growth inhibitory effects in the invasive human bladder cancer cell line, T24, and that this anti-tumour effect might be mediated, at least in part, by reversing EMT.

In conclusion, we demonstrated that etodolac at a dose comparable with that used clinically could induce *in vitro* and *in vivo* anti-tumour effects and a reversal effect on the EMT in the invasive bladder cancer cell line T24. The reversal effect on the EMT was assumed based on the observation that etodolac markedly reduced the *SNAIL* mRNA expression and the vimentin cell surface expression and induced the *E-cadherin* mRNA expression and the E-cadherin cell surface expression, and also strongly inhibited cell migration in T24 cells. Our findings suggest that etodolac is a good candidate for an anti-tumour or chemopreventive reagent for high-grade bladder cancer that has a high risk of recurrence and progression to invasion.
